# Supplementary figures and images for: Global expansion and redistribution of Aedes-borne virus transmission risk with climate change
Source: PLoS Negl Trop Dis. 2019 Mar 28;13(3):e0007213. doi: 10.1371/journal.pntd.0007213 (PMC6438455; doi:10.1371/journal.pntd.0007213)

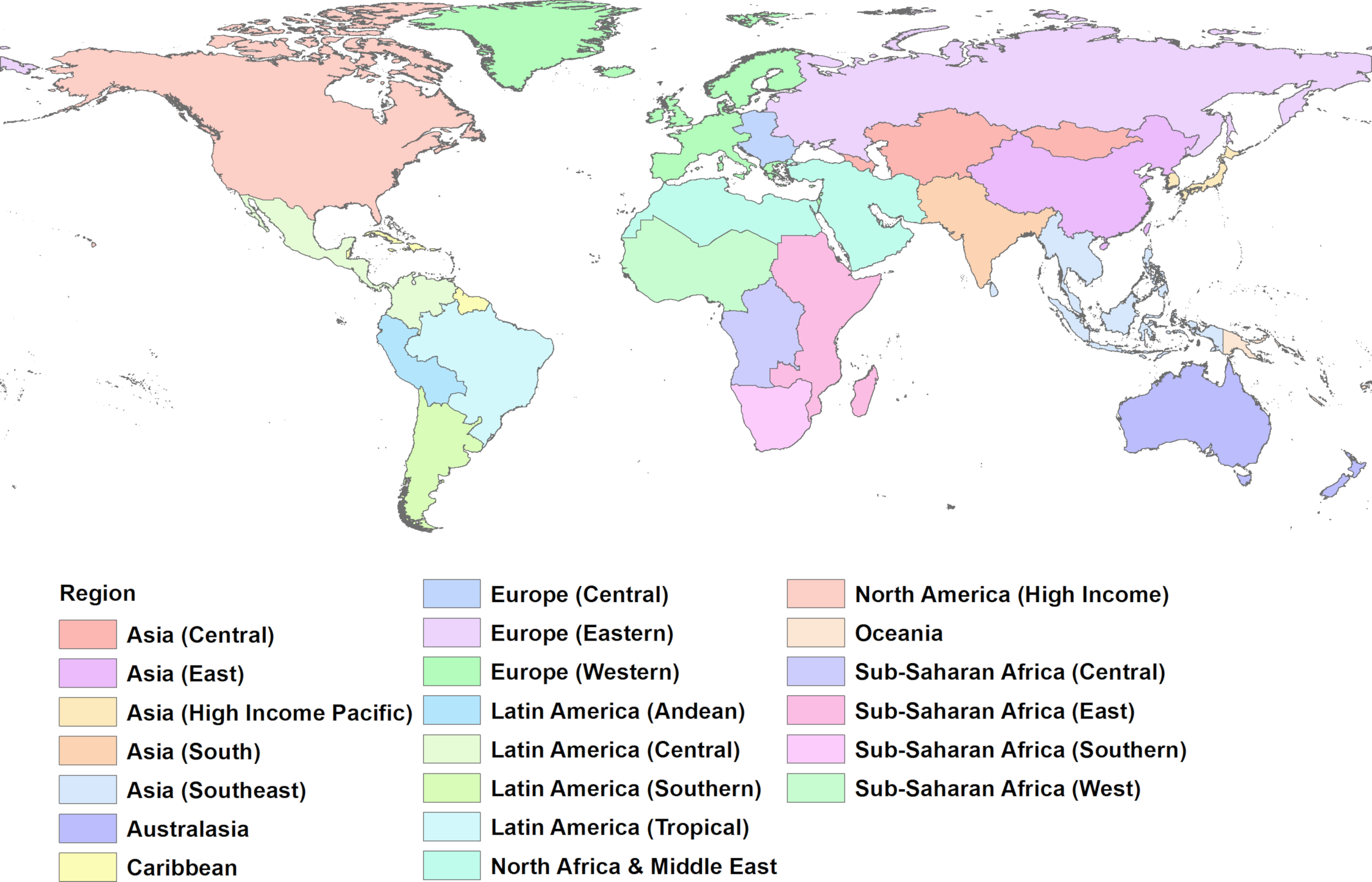

Supplement: S1 Fig — We adopt the same system as the Global Burden of Disease Study in our regional breakdown. (TIF) [file pntd.0007213.s001.tif]
