# Supplementary material for: Global expansion and redistribution of Aedes-borne virus transmission risk with climate change
Source: PLoS Negl Trop Dis. 2019 Mar 28;13(3):e0007213. doi: 10.1371/journal.pntd.0007213 (PMC6438455; doi:10.1371/journal.pntd.0007213)
Supplement: S1 Table — All values are given in millions; future projections are averaged across GCMs, broken down by year (2050, 2080) and RCP (2.6, 4.5, 6.0, 8.5), and are given as net change from current population at risk. 0+/0- denote the sign of smaller non-zero values that rounded to 0.0, whereas “0” denotes true zeros. (Losses do not indicate loss of any transmission, only to reduction 11 or fewer months.). (DOCX) [file pntd.0007213.s002.docx]

**S1 Table. Changing year-round (12 month) population at risk due to temperature suitability for *Aedes aegypti* virus transmission*.*** All values are given in millions; future projections are averaged across GCMs, broken down by year (2050, 2080) and RCP (2.6, 4.5, 6.0, 8.5), and are given as net change from current population at risk. 0+/0- denote the sign of smaller non-zero values that rounded to 0.0, whereas “0” denotes true zeros. (Losses do not indicate loss of any transmission, only to reduction 11 or fewer months.).

| **Region** | **Current** | **2050** | | | | **2080** | | | |
| --- | --- | --- | --- | --- | --- | --- | --- | --- | --- |
|  |  | **2.6** | **4.5** | **6.0** | **8.5** | **2.6** | **4.5** | **6.0** | **8.5** |
| Asia (Central) | 0 | 0 | 0 | 0 | 0 | 0 | 0 | 0 | 0 |
| Asia (East) | 0+ | 1.4 | 1.8 | 1.1 | 3.7 | 1.6 | 4.5 | 4 | 8.3 |
| Asia (High Income Pacific) | 3.6 | -0.2 | -0.2 | -0.2 | -0.2 | -0.2 | -0.2 | -0.2 | -0.2 |
| Asia (South) | 286.4 | 21.8 | 71.8 | 13.7 | 73.6 | 12.1 | 89.7 | 72.6 | 29.6 |
| Asia (Southeast) | 499.4 | 19.2 | 22.4 | 19.9 | 25.1 | 18.9 | 26.3 | 15.4 | -10.3 |
| Australasia | 0.2 | 0+ | 0+ | 0+ | 0.1 | 0+ | 0.2 | 0.2 | 0.3 |
| Caribbean | 34.8 | 1.8 | 2.2 | 2.1 | 2.8 | 1.7 | 2.6 | 2.9 | 3.3 |
| Europe (Central) | 0 | 0 | 0 | 0 | 0 | 0 | 0 | 0 | 0 |
| Europe (Eastern) | 0 | 0 | 0 | 0 | 0 | 0 | 0 | 0 | 0 |
| Europe (Western) | 0 | 0 | 0 | 0 | 0 | 0 | 0 | 0 | 0+ |
| Latin America (Andean) | 14.0 | 3.9 | 4.8 | 4.6 | 5.7 | 3.5 | 5.4 | 5.8 | 7.5 |
| Latin America (Central) | 88.1 | 13.0 | 18.8 | 17.0 | 25.8 | 12.0 | 24.4 | 27.4 | 34.1 |
| Latin America (Southern) | 0 | 0 | 0 | 0 | 0 | 0 | 0 | 0 | 0.2 |
| Latin America (Tropical) | 67.5 | 27.2 | 34.5 | 30.8 | 41.5 | 27.3 | 39 | 42.9 | 54.9 |
| North Africa & Middle East | 12.5 | -5.2 | -5.5 | -6.0 | -5.6 | -4.7 | -5.4 | -5.4 | -3.9 |
| North America (High Income) | 0.5 | 0.3 | 0.9 | 0.6 | 1.5 | 0.3 | 1.9 | 1.6 | 5.5 |
| Oceania | 0 | 0 | 0 | 0 | 0 | 0 | 0 | 0 | 0 |
| Sub-Saharan Africa (Central) | 5.3 | 0.3 | 0.6 | 0.4 | 0.9 | 0.3 | 0.7 | 0.9 | 1.7 |
| Sub-Saharan Africa (East) | 79.0 | 19.1 | 23.1 | 22.4 | 26.8 | 16.6 | 25.4 | 28.0 | 36.1 |
| Sub-Saharan Africa (Southern) | 126.9 | 43.8 | 60.7 | 56.7 | 78.3 | 37.9 | 74.3 | 85.5 | 110.3 |
| Sub-Saharan Africa (West) | 0 | 0+ | 0.1 | 0.1 | 0.3 | 0+ | 0.2 | 0.6 | 4.4 |
