# Supplementary material for: Global expansion and redistribution of Aedes-borne virus transmission risk with climate change
Source: PLoS Negl Trop Dis. 2019 Mar 28;13(3):e0007213. doi: 10.1371/journal.pntd.0007213 (PMC6438455; doi:10.1371/journal.pntd.0007213)
Supplement: S2 Table — All values are given in millions; future projections are averaged across GCMs, broken down by year (2050, 2080) and RCP (2.6, 4.5, 6.0, 8.5), and are given as net change from current population at risk. 0+/0- denote the sign of smaller non-zero values that rounded to 0.0, whereas “0” denotes true zeros. (Losses do not indicate loss of any transmission, only to reduction 11 or fewer months). (DOCX) [file pntd.0007213.s003.docx]

**S2 Table. Changing year-round (12 month) population at risk due to temperature suitability for *Aedes albopictus* virus transmission *.*** All values are given in millions; future projections are averaged across GCMs, broken down by year (2050, 2080) and RCP (2.6, 4.5, 6.0, 8.5), and are given as net change from current population at risk. 0+/0- denote the sign of smaller non-zero values that rounded to 0.0, whereas “0” denotes true zeros. (Losses do not indicate loss of any transmission, only to reduction 11 or fewer months).

| **Region** | **Current** | **2050** | | | | **2080** | | | | |
| --- | --- | --- | --- | --- | --- | --- | --- | --- | --- | --- |
|  |  | **2.6** | **4.5** | **6.0** | **8.5** | | **2.6** | **4.5** | **6.0** | **8.5** |
| Asia (Central) | 0 | 0 | 0 | 0 | 0 | | 0 | 0 | 0 | 0 |
| Asia (East) | 1.3 | 1.4 | -0.4 | -0.5 | -1 | | 1 | -0.9 | -1 | -1.2 |
| Asia (High Income Pacific) | 3.6 | -0.3 | -0.5 | -0.4 | -2.9 | | -0.2 | -2.2 | -3.1 | -3.6 |
| Asia (South) | 98.3 | -73 | -80.3 | -78.9 | -87.3 | | -67.7 | -86.1 | -88.5 | -92.6 |
| Asia (Southeast) | 435.3 | -133.9 | -213.3 | -190.9 | -277.4 | | -131.9 | -254.8 | -282.7 | -343.6 |
| Australasia | 0.2 | 0+ | 0+ | 0+ | 0- | | 0+ | 0- | 0- | 0- |
| Caribbean | 39.3 | -5.9 | -11.7 | -9.4 | -17.5 | | -4.5 | -16.1 | -18.1 | -28.0 |
| Europe (Central) | 0 | 0 | 0 | 0 | 0 | | 0 | 0 | 0 | 0 |
| Europe (Eastern) | 0 | 0 | 0 | 0 | 0 | | 0 | 0 | 0 | 0 |
| Europe (Western) | 0 | 0 | 0 | 0 | 0+ | | 0 | 0+ | 0+ | 0.1 |
| Latin America (Andean) | 17.9 | 2 | 0.1 | 0.5 | -3 | | 1.8 | -2 | -3.1 | -5 |
| Latin America (Central) | 97.2 | -23.2 | -26.8 | -25.3 | -31.0 | | -20.8 | -29.3 | -31.4 | -33.6 |
| Latin America (Southern) | 0 | 0 | 0 | 0 | 0+ | | 0 | 0 | 0+ | 0+ |
| Latin America (Tropical) | 93.6 | -0.8 | -5.2 | -6.1 | -9.7 | | -2.9 | -10.1 | -12.9 | -37.1 |
| North Africa & Middle East | 2.6 | 0+ | 0+ | -0.2 | -0.2 | | 0.1 | 0- | -0.2 | -0.1 |
| North America (High Income) | 1 | 2.8 | 1.4 | 1 | 0+ | | 1.6 | 0.1 | -0.2 | -0.2 |
| Oceania | 0 | 0 | 0 | 0 | 0 | | 0 | 0 | 0 | 0 |
| Sub-Saharan Africa (Central) | 5.9 | 0.4 | 0.4 | 0.5 | 0.1 | | 0.4 | 0.1 | 0- | -1.4 |
| Sub-Saharan Africa (East) | 96.6 | 8.0 | 5.1 | 6.8 | -4.5 | | 7.5 | -9.1 | -9.9 | -45.8 |
| Sub-Saharan Africa (Southern) | 133.5 | 31.9 | 38.8 | 38.8 | 43.4 | | 29.7 | 39.5 | 43.9 | 39.2 |
| Sub-Saharan Africa (West) | 0+ | 0+ | 0.5 | 0.4 | 1.8 | | 0+ | 0.9 | 2 | 6.2 |
