# Supplementary material for: Global expansion and redistribution of Aedes-borne virus transmission risk with climate change
Source: PLoS Negl Trop Dis. 2019 Mar 28;13(3):e0007213. doi: 10.1371/journal.pntd.0007213 (PMC6438455; doi:10.1371/journal.pntd.0007213)
Supplement: S3 Table — Regions are ranked based on millions of people exposed for the first time to any transmission risk; parentheticals give the net change (first exposures minus populations escaping transmission risk). All values are given for the worst-case scenario (RCP 8.5) in the longest term (2080). (DOCX) [file pntd.0007213.s004.docx]

**Table S3. Top 10 regional increases in populations experiencing year-round temperature suitability for transmission (12 months).** Regions are ranked based on millions of people exposed for the first time to any transmission risk; parentheticals give the net change (first exposures minus populations escaping transmission risk). All values are given for the worst-case scenario (RCP 8.5) in the longest term (2080).

| ***Aedes aegypti*** | | ***Aedes albopictus*** | |
| --- | --- | --- | --- |
| 1. Asia (South) | 209.9 (29.6) | 1. Sub-Saharan Africa (East) | 114.3 (39.2) |
| 2. Sub-Saharan Africa (East) | 152.6 (110.3) | 2. Latin America (Tropical) | 39.7 (-37.1) |
| 3. Latin America (Tropical) | 63.2 (54.9) | 3. Latin America (Central) | 38.1 (-33.6) |
| 4. Asia (Southeast) | 44 (-10.3) | 4. Sub-Saharan Africa (Central) | 23.1 (-45.8) |
| 5. Latin America (Central) | 40.7 (34.1) | 5. Asia (Southeast) | 16.3 (-343.6) |
| 6. Sub-Saharan Africa (Central) | 36.6 (36.1) | 6. Latin America (Andean) | 8.6 (-5) |
| 7. Sub-Saharan Africa (West) | 8.7 (-130.2) | 7. Sub-Saharan Africa (Southern) | 6.2 (6.2) |
| 8. Asia (East) | 8.3 (8.3) | 8. Sub-Saharan Africa (West) | 2.5 (-194) |
| 9. Latin America (Andean) | 8 (7.5) | 9. North Africa & Middle East | 2.4 (-0.1) |
| 10. North Africa & Middle East | 7.4 (-3.9) | 10. Oceania | 2 (-1.4) |
| **Total** (across all 21 regions) | **597.2 (151.6)** | **Total** (across all 21 regions) | **256.5 (-740.8)** |
